# RA-UNet: A Hybrid Deep Attention-Aware Network to Extract Liver and Tumor in CT Scans

**DOI:** 10.3389/fbioe.2020.605132

**Published:** 2020-12-23

**Authors:** Qiangguo Jin, Zhaopeng Meng, Changming Sun, Hui Cui, Ran Su

**Affiliations:** ^1^School of Computer Software, College of Intelligence and Computing, Tianjin University, Tianjin, China; ^2^CSIRO Data61, Sydney, NSW, Australia; ^3^Tianjin University of Traditional Chinese Medicine, Tianjin, China; ^4^Department of Computer Science and Information Technology, La Trobe University, Melbourne, VIC, Australia

**Keywords:** medical image segmentation, tumor segmentation, u-net, residual learning, attention mechanism

## Abstract

Automatic extraction of liver and tumor from CT volumes is a challenging task due to their heterogeneous and diffusive shapes. Recently, 2D deep convolutional neural networks have become popular in medical image segmentation tasks because of the utilization of large labeled datasets to learn hierarchical features. However, few studies investigate 3D networks for liver tumor segmentation. In this paper, we propose a 3D hybrid residual attention-aware segmentation method, i.e., RA-UNet, to precisely extract the liver region and segment tumors from the liver. The proposed network has a basic architecture as U-Net which extracts contextual information combining low-level feature maps with high-level ones. Attention residual modules are integrated so that the attention-aware features change adaptively. This is the first work that an attention residual mechanism is used to segment tumors from 3D medical volumetric images. We evaluated our framework on the public MICCAI 2017 Liver Tumor Segmentation dataset and tested the generalization on the 3DIRCADb dataset. The experiments show that our architecture obtains competitive results.

## 1. Introduction

**Figure 1:**
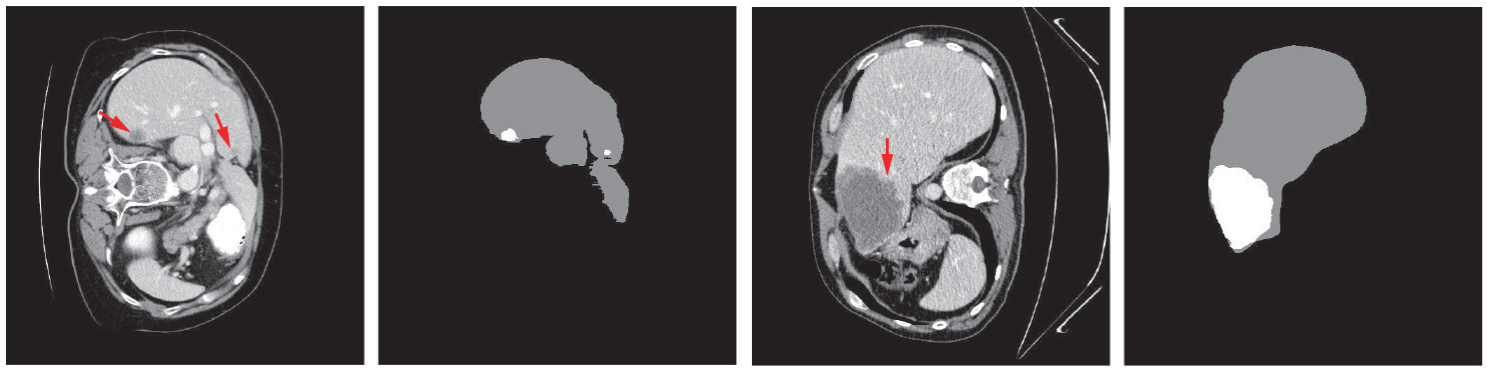
Examples of typical 2D CT scans and the corresponding ground truth of liver/tumor extractions where red arrows indicate the tumor/lesion regions. The typical scans are from the MICCAI 2017 Liver Tumor Segmentation (LiTS) dataset.

Liver tumors, or hepatic tumors, are great threats to human health. The malignant tumor, also known as the liver cancer, is one of the most frequent internal malignancies worldwide (6%), and is also one of the leading death causes from cancer (9%) (WHO, 2014a,b). Even the benign (non-cancerous) tumors may grow large enough to cause health problems. Computed tomography (CT) is used to assist the diagnosis of liver tumors (Christ et al., 2017a). The extraction of liver and tumors from CT is a critical task before surgical intervention in choosing an optimal approach for treatment. Accurate segmentation of liver and tumor from medical images provides their precise locations in the human body. Then therapies evaluated by the specialists can be provided to treat individual patients (Rajagopal and Subbaiah, 2015). However, due to the heterogeneous and diffusive shapes of liver and tumor, segmenting them from CT images is challenging. Numerous efforts have been taken to tackle the segmentation task on liver/tumors. Figure 1 shows some typical liver and tumor CT scans.

In general, liver and tumor extraction approaches can be classified into three categories: manual segmentation, semi-automated segmentation, and automated segmentation. Manual segmentation is a subjective, poorly reproducible, and time-consuming approach. It heavily depends upon human recognizable features, and requires people with high-level technical skills. These factors make it impractical for real applications (Li et al., 2015). Semi-automated segmentation requires initial human intervention, which may cause bias and mistakes. In order to accelerate and facilitate diagnosis, therapy planning, and monitoring, and finally help surgeons remove tumors, it is necessary to develop an automated and precise method to segment tumors from CT images. However, the large scale spatial and structural variability, low contrast between liver and tumor regions, existence of noise, partial volume effects, complexity of 3D-spatial tumor features, or even the similarity between nearby organs make the automation of segmentation quite a difficult task (Li et al., 2015). Recently, convolutional neural networks (CNN) have been applied to many volumetric image segmentations. A number of CNN models including both 2D and 3D networks have been developed. However, the 3D networks are usually not as efficient and flexible as the corresponding 2D networks. For instance, 2D and 3D fully convolutional networks (FCNs) have been proposed for semantic segmentation (Long et al., 2015). Yet due to the high computational cost and the low efficiency of 3D convolutions, the depth of the 3D FCNs is limited compared to that of 2D FCNs, which makes it impractical for 2D networks to be extended to 3D networks.

To address these issues and inspired by the residual networks (He et al., 2016) and the attention residual learning (Wang et al., 2017), we propose a hybrid residual attention-aware liver and tumor extraction neural network named RA-UNet^1^, which is designed to effectively extract 3D volumetric contextual features of liver and tumor from CT images in an end-to-end manner. The proposed network integrates a residual U-Net architecture and an attention residual learning mechanism which enables the optimization and performance improvement on deep networks. The contributions of our works are listed as follows: Firstly, the attention mechanism can have the capability of focusing on specific parts of the image. Different types of attention are possible through stacking attention modules so that the attention-aware features can change adaptively. Secondly, we use the 3D U-Net as the basic architecture to capture multi-scale attention information and to integrate low-level features with high-level ones. Besides, RA-UNet, which directly segments the liver and tumor from 3D medical volumes, enlarges the U-Net family in 3D medical image analysis. What's more, our model does not depend on any pre-trained model or commonly used post processing techniques, such as 3D conditional random fields. The generalization of the proposed approach is demonstrated through testing on the 3DIRCADb dataset (Soler et al., 2010). Our architecture achieves competitive performances comparing with other state-of-the-art methods on the MICCAI 2017 Liver Tumor Segmentation (LiTS) dataset, and also shows high generalization. Our paper is organized as follows. In section 2, we briefly review the state-of-the-art automated liver tumor segmentation methods. We illustrate the methodologies in detail including the datasets, preprocessing strategy, hybrid deep learning architecture, and training procedure in section 3. In section 4, we evaluate the proposed algorithm, report the experimental results, compare with some other approaches, and extend our approach to other medical segmentation tasks. Conclusions and future works are given in section 5.

## 2. Related Works

In the past decades, various applications have been developed via computer-aided methods in medical/biomedical image processing, cellular biology domains (Zeng et al., 2017; Hong et al., 2020a,b; Song et al., 2020a,b, 2021). Recently, with the advance of artificial intelligence, deep learning has been used in a number of areas such as natural language processing, anti-cancer drug response prediction, and image analysis (Liu et al., 2017; Su et al., 2019; Zeng et al., 2020). Some have achieved state-of-the-art performances in medical imaging challenges (Litjens et al., 2017; Jin et al., 2019).

### 2.1. Deep Learning in Medical Image Analysis

Unlike the traditional methods that use hand-crafted features, deep neural networks (DNNs) are able to automatically learn discriminative features. The learned features which contain hierarchical information have the ability to represent each level of the input data. Among those methods, CNN is one of the most popular methods and has shown impressive performance for 3D medical image analysis tasks. Multi-scale patch-based and pixel-based strategies were proposed to improve the segmentation performance. For instance, Zhang et al. (2015) proposed a method which used a deep CNN for segmenting brain tissues using multi-modality magnetic resonance images (MRI). Li et al. (2015) presented an automatic method based on 2D CNN to segment lesions from CT slices and compared the CNN model with other traditional machine learning techniques, which included AdaBoost (Collins et al., 2002), random forests (RF) (Breiman, 2001), and support vector machine (SVM) (Furey et al., 2000). This study showed that CNN still had limitations on segmenting tumors with uneven densities and unclear borders. Pereira et al. (2016) proposed a CNN architecture with small kernels for segmenting brain tumors on MRI. This architecture reached Dice similarity coefficient metrics of 0.78, 0.65, and 0.75 for the complete, core, and enhancing regions respectively. Lee et al. (2011) presented a CNN-based architecture that could learn from provided labels to construct brain segmentation features. However, due to low memory requirements, low complexity of computation, and lots of pre-trained models, most of the latest CNN architectures including the methods reviewed above used 2D slices from 3D volumes for carrying out the segmentation task. However, the spatial structural organizations of organs are not considered, and the volumetric information is not fully utilized. Therefore, 3D automatic segmentation which makes full use of spatial information is urgently needed for surgeons.

### 2.2. 3D Convolutional Neural Networks

In order to sufficiently add 3D spatial structures into CNN for 3D medical image analysis, 3D CNN which considers axial direction of the 3D volumes has recently been proposed in medical imaging field. Shakeri et al. (2016) proposed a 2D CNN architecture to detect tumors from a set of brain slices. Then they additionally applied a 3D conditional random field (CRF) algorithm for post processing in order to impose volumetric homogeneity. This is one of the earliest studies that used CNN-related segmentation on volumetric images. Çiçek et al. (2016) learned from sparsely sequential volumetric images by feeding a U-Net with 2D sequential slices. 3D CNN-based segmentation methods were then employed in a large scale. Andermatt et al. (2016) used a 3D recurrent neural network (RNN) with gated recurrent units to segment gray and white matters in a brain MRI dataset. Dolz et al. (2017) investigated a 3D FCN for subcortical brain structure segmentation in MRI images. They reduced the computational and memory costs, which were quite severe issues for 3D CNN, via small kernels with a deeper network. Bui et al. (2017) proposed a deep densely convolutional network for volumetric brain segmentation. This architecture provided a dense connection between layers. They concatenated feature maps from fine and coarse blocks, which allowed to capture multi-scale contextual information. The 3D deeply supervised network (DSN), which had a much faster convergence and better discrimination capability, could be extended to other medical applications (Dou et al., 2016). Oktay et al. (2018) proposed a novel attention gate model called attention U-Net for medical imaging which could learn to concentrate on target structures of different shapes and sizes. However, due to hardware limitations, 3D convolutional medical image segmentation is still a bottleneck.

### 2.3. Liver Tumor Segmentation

As for liver tumor detection in 3D volumetric images, not many explorations have been made using the CNN-based methods. Lu et al. proposed a method based on 3D CNN to carry out the probabilistic segmentation task and used graph cut to refine the previous segmentation result. However, as tested only on one dataset, the generality of this architecture still needs to be validated (Lu et al., 2017). Christ et al. (2017a) proposed a cascaded FCNs (CFCNs) to segment liver and its lesions in CT and MRI images, which enabled segmentation for large scale medical trials. They trained the first FCN to segment the liver and trained the second FCN to segment its lesions based on the predicted liver region of interest (ROI). This approach reached a Dice score of 94%. Additionally, Christ et al. (2017b) also predicted hepatocellular carcinoma (HCC) malignancy using two CNN architectures. They took a CFCN as the first step to segment tumor lesions. Then they applied a 3D neural network called SurvivalNet to predict the lesions' malignancy. This method achieved an accuracy of 65% with a Dice score of 69% for lesion segmentation and an accuracy of 68% for tumor malignancy detection. Kaluva et al. (2018) proposed a fully automatic 2-stage cascaded method for liver and tumor segmentation based on the LiTS dataset, and they reached global Dice scores of 0.923 and 0.623 on liver and tumor, respectively. Bi et al. (2017) integrated 2D residual blocks into their network and gained a Dice score of 0.959. Moreover, Li et al. (2018) built a hybrid densely connected U-Net for liver and tumor segmentation, which combined both 2D and 3D features on liver and tumor. They reached Dice scores of 0.961 and 0.722 on liver and tumor segmentation, respectively. Pandey et al. (2018) reduced the complexity of a deep neural network by introducing ResNet-blocks and obtained a Dice score of 0.587 on tumor segmentation. Recently, Tang et al. (2020) proposed a two-stage framework for 2D liver and tumor segmentation. The proposed network explicitly captured complementary objects (liver and tumor) and their edge information to preserve the organ and lesion boundaries. Heker and Greenspan (2020) introduced transfer learning and joint learning to improve the network's generalization and robustness for liver lesion segmentation and classification. Seo et al. (2019) modified the U-Net with Object-Dependent high-level features for the liver tumor segmentation challenge. However, as mentioned earlier, most of them segmented the liver or lesion regions based on 2D slices from 3D volumes. The spatial information has not been taken into account to the maximum extent.

Recently, attention based image classification (Wang et al., 2017) and semantic segmentation architectures (Chen et al., 2016) have attracted a lot of attention. Some medical imaging tasks have used the attention mechanism to solve the issues in real applications. For instance, Schlemper et al. (2019) proposed an attention-gated networks for real-time automated scan plane detection in fetal ultrasound screening. The integrated self-gated soft-attention mechanisms, which can be easily incorporated into other networks, achieved good performances. Overall, it is expected that 3D deep networks combined with the attention mechanism would achieve a good performance for liver/tumor extraction tasks.

## 3. Methodology

### 3.1. Overview of Our Proposed Architecture

**Figure 2:**
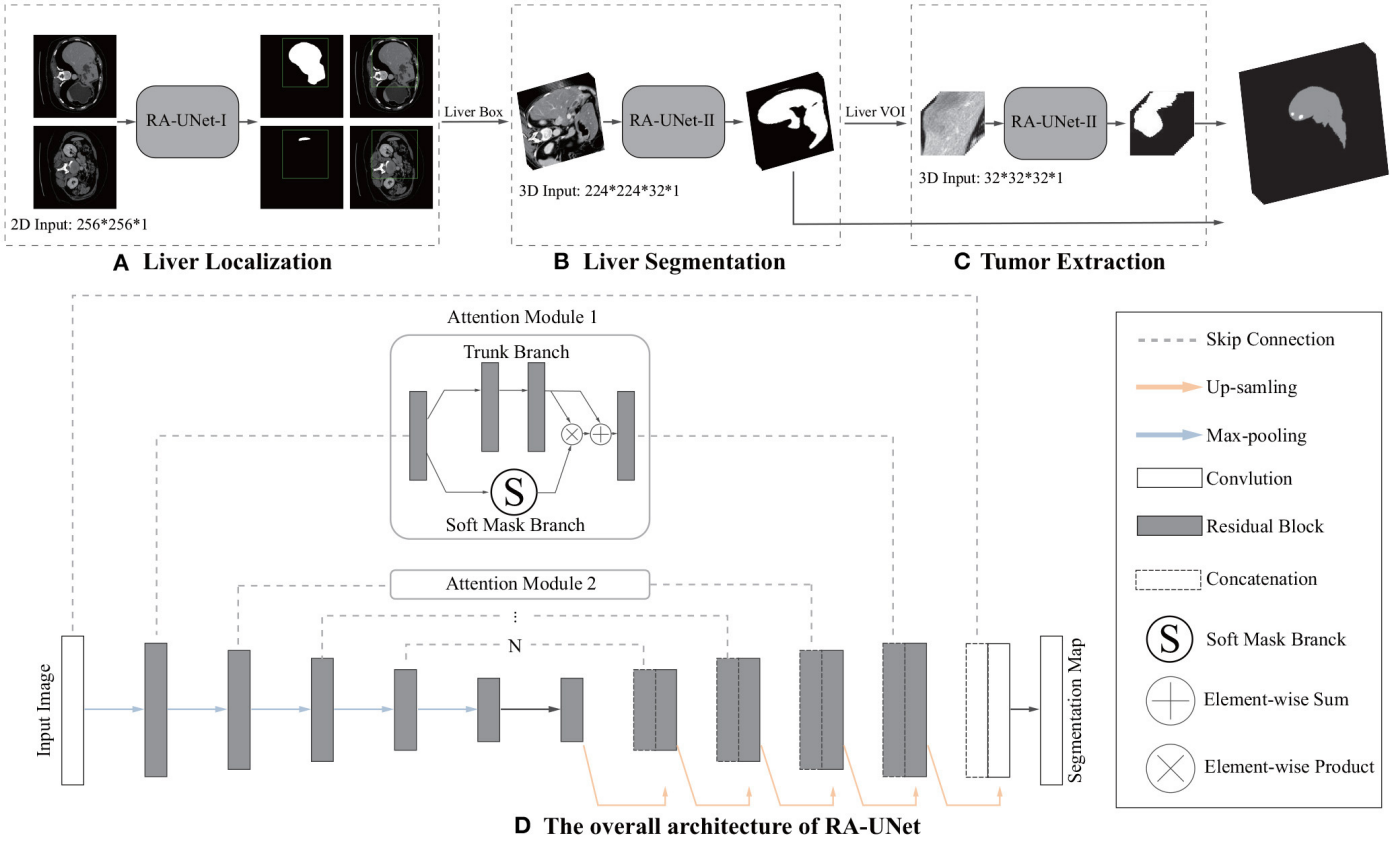
Overview of the proposed pipeline of liver and tumor segmentation. **(A)** A simple version of 2D RA-UNet (RA-UNet-I) is employed for coarse localization of a liver region within a boundary box. **(B)** The 3D RA-UNet (RA-UNet-II) is designed for hierarchically extracting attention-aware features of liver volume of interest (VOI) inside the liver boundary box. **(C)** RA-UNet-II is responsible for an accurate tumor extraction which is inside the liver VOI. **(D)** The overall architecture of RA-UNet.

The first time that an attention mechanism was introduced in semantic image segmentation was in Chen et al. (2016), which combined *share-net* with attention mechanisms and achieved good performances. More recently, the attention mechanism is gradually applied to medical image segmentation (Oktay et al., 2018; Schlemper et al., 2019). Inspired by residual attention learning (Wang et al., 2017) and U-Net (Ronneberger et al., 2015), we propose the RA-UNet that for the liver and tumor segmentation tasks. Our overall architecture for segmentation is depicted in Figure 2. The proposed architecture consists of three main stages which extract liver and tumor sequentially. Firstly, in order to reduce the overall computational time, we used a 2D residual attention-aware U-Net (RA-UNet) named RA-UNet-I to obtain a coarse liver boundary box. Next, a 3D RA-UNet, which is called RA-UNet-II, was trained to obtain a precise liver volume of interest (VOI). Finally, the obtained liver VOI was sent to a second RA-UNet-II to extract the tumor region. The designed network can handle volumes in various complicated conditions and obtain desirable results in different liver/tumor datasets.

### 3.2. Datasets and Materials

In our study, we used the public Liver Tumor Segmentation Challenge (LiTS) dataset to evaluate the proposed architecture. This dataset has a total of 200 CT scans containing 130 scans as training data and 70 scans as test data, both of which have the same 512 × 512 in-plane resolution but with different numbers of axial slices in each scan. These training data and their corresponding ground truth are provided by various clinical sites around the world, while the ground truth of the test data is not available.

Another dataset named 3DIRCADb is used as an external test dataset to validate the generalization and scalability of our model. It includes 20 enhanced CT scans and the corresponding manually segmented tumors from European hospitals. The number of axial slices, which have 512 × 512 in-plane resolution, differs for each scan.

### 3.3. Data Preprocessing

For a medical image volume, Hounsfield units (HU) is a measurement of relative densities determined by CT. Normally, the HU values range from −1,000 to 1,000. Because tumors grow on the liver tissue, the surrounding bones, air, or irrelevant tissues may disturb the segmentation result. Hence, an initial segmentation was used to filter out those noises, leaving the liver region clean which is yet to be segmented. In terms of convenience and efficiency, we took a global windowing step as our data preprocessing strategy.

We list the typical radiodensities of some main tissues in Table 1, which shows that these tissues have a wide range of HU values. From the table, the HU value for air is typically above −200; for bone, it is the highest HU values among these tissues; for liver, it is from 40 to 50 HU; for water, it is approximately from 0 to 10 HU; and for blood, it is from 3 to 14 HU.

**Table 1:** Typical tissues radiodensities of human body.

**Tissue**	**HU**
Air	−200+
Bone	400+
Liver	40~50
Water	0 ± 10
Blood	3~14

**Figure 3:**
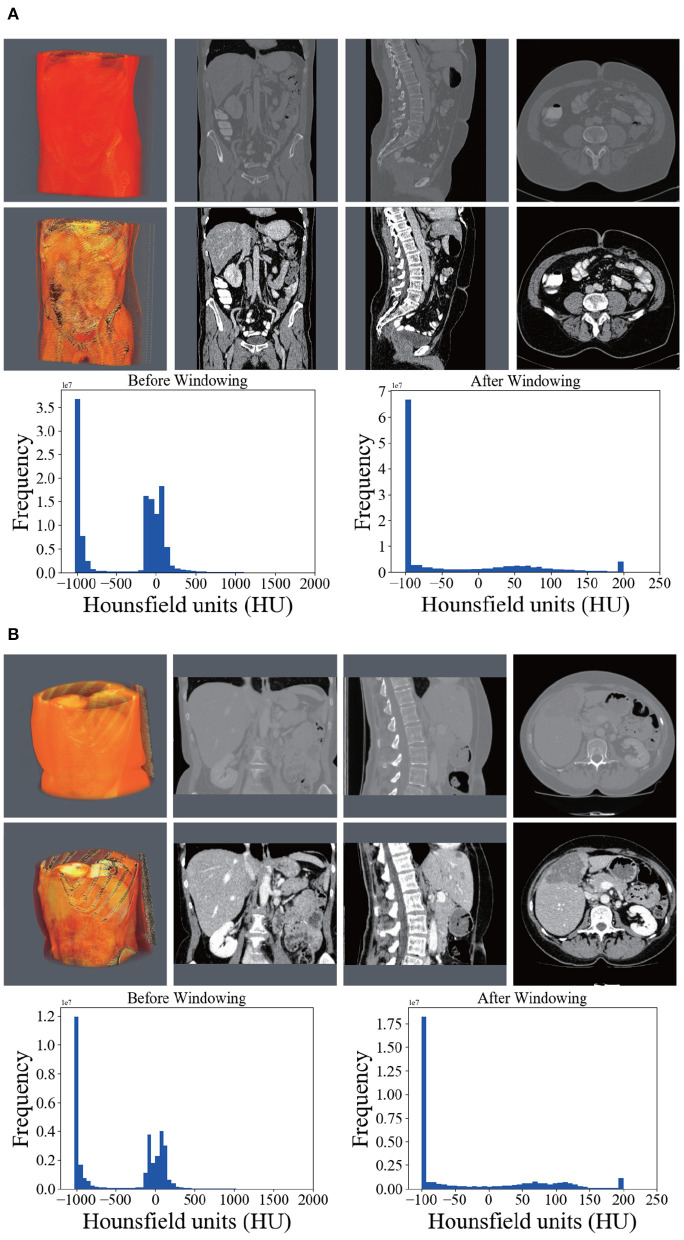
Comparison between the raw CT scans (first row), windowed (second row) scans, and histograms of HU (third row) before and after windowing. **(A)** Shows the comparison on LiTS. **(B)** Shows the comparison on 3DIRCADb.

In this article, we set the HU window at the range from −100 to 200. With such a window, irrelevant organs and tissues were mostly removed. The first rows of Figure 3 shows the 3D, coronal, sagittal, and axial plane views of the raw volumes of LiTS and 3DIRCADb, respectively. The second rows show the preprocessed volumes with irrelevant organ removed. It can be seen that most of the noise has been removed. The distribution of HU values before and after windowing is illustrated on the left and right of the third rows in Figure 3 where Frequency denotes the frequency of HU values. We applied the zero-mean normalization and min-max normalization on the data after the windowing. No further image processing was performed.

### 3.4. RA-UNet Architecture

#### 3.4.1. U-Net as the Basic Architecture

Our RA-UNet has an overall architecture similar to the standard U-Net, consisting of an encoder and a decoder symmetrically on the two sides of the architecture. The contextual information is propagated by the encoder within the rich skip connections which enables the extraction of hierarchical features with more complexities. The decoder receives features that have diverse complexities and reconstructs the features in a coarse-to-fine manner. An advantage is that the U-Net introduces long-range connections through the encoder part and the corresponding decoder part, so that different hierarchical features from the encoder can be merged to the decoder which makes the network much more precise and expansible.

#### 3.4.2. Residual Learning Mechanism

The network depth is of crucial importance. However, gradient vanishing is a common problem in a very deep neural network when carrying out back propagation, which results in poor training results. In order to overcome this problem, He et al. proposed the deep residual learning framework to learn the residual of the identity map (He et al., 2016). In our study, residual blocks are stacked except the first layer and the last layer (Figure 2D) to unleash the capability of deep neural networks. The stacked residual blocks solve the gradient vanishing problem at the structural level of the neural network by using identity mappings as the skip connections. The residual units directly propagate features from early convolution to late convolution and consequently improve the performance of the model. The residual block is defined as:

(1) $$\begin{array}{l} {\text{OR}}_{i,c}\left(\text{x}\right)=\text{x}+{\text{f}}_{i,c}\left(\text{x}\right) \end{array}$$

where **x** denotes the first input of a residual block, ***OR*** denotes the output of a residual block, *i* ranges over all spatial positions, *c* ∈ {1, …, *C*} indicates the index of channels, *C* is the total number of channels, and ***f*** represents the residual mapping to be learned.

**Figure 4:**
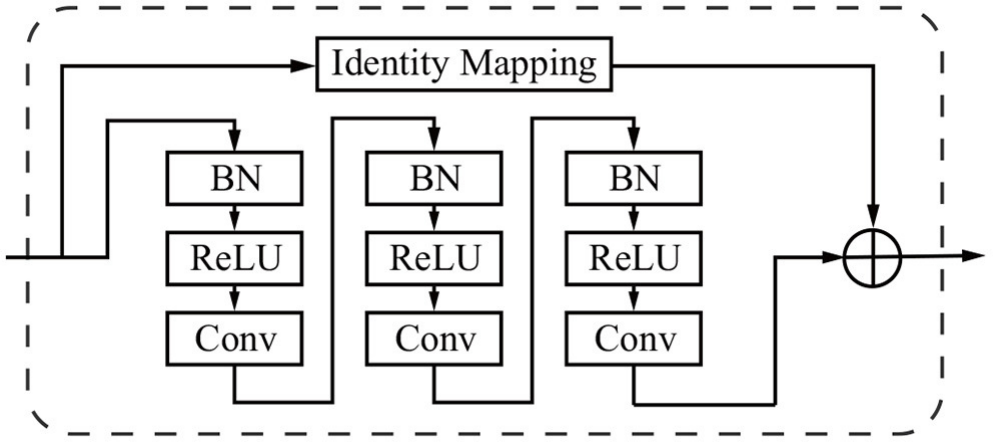
Sample of a residual block in the dashed window. An identity mapping and convolutional blocks are added before the final feature output.

The residual block consists of three sets of combinations of a batch normalization (BN) layer, an activation (ReLU) layer, and a convolutional layer. A convolutional identity mapping connection is used to ensure the accuracy as the network goes “deeper” (He et al., 2016). The detailed residual unit is illustrated in Figure 4.

#### 3.4.3. Attention Residual Learning Mechanism

The performance will drop if only naive stacking is used for the attention modules. This can be solved by the attention residual learning proposed by Wang et al. (2017). The attention residual mechanism divides the attention module into a trunk branch and a soft mask branch, where the trunk branch is used to process the original features and the soft mask branch is used to construct the identity mapping. The output ***OA*** of the attention module under attention residual learning can be formulated as:

(2) $$\begin{array}{l} {\text{OA}}_{i,c}\left(\text{x}\right)=\left(1+{\text{S}}_{i,c}\left(\text{x}\right)\right){\text{F}}_{i,c}\left(\text{x}\right) \end{array}$$

where ***S***(***x***) has values in [0,1]. If ***S***(***x***) is close to 0, ***OA***(***x***) will approximate the original feature maps ***F***(***x***). The soft mask branch ***S***(***x***), which selects identical features and suppresses noised from the trunk branch, plays the most important role in the attention residual mechanism.

**Figure 5:**
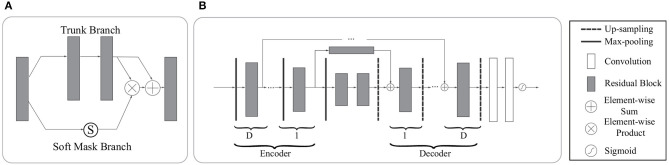
The architecture of the attention residual module. **(A)** The attention residual module contains a trunk branch and a soft mask branch. The trunk branch learns original features while the soft mask branch focuses on reducing noises and enhancing good features. **(B)** The soft mask branch contains a stack of encoder-decoder blocks. D denotes the depth of skip connections. In our network, we set D to 0,1,2,3 according to the specific location of the attention residual block.

The soft mask branch has an encoder-decoder structure which has been widely applied to medical image segmentation (Ronneberger et al., 2015; Çiçek et al., 2016; Alom et al., 2018). In the attention residual mechanism, it is designed to enhance good features and reduce the noises from the trunk branch. The encoder in the soft mask branch contains a max-pooling operation, a residual block, and a long-range residual block connected to the corresponding decoder, where an element-wise sum is performed following a residual block and an up-sampling operation. After the encoder and decoder parts of the soft mask, two convolutional layers and one Sigmoid layer are added to normalize the output. Figure 5 illustrates the attention residual module in detail.

In general, the attention residual mechanism can keep the original feature information through the trunk branch and pay attention to those liver tumor features by the soft mask branch.

#### 3.4.4. Loss Function

The weights are learnt by minimizing the loss function. We employed a loss function based on the Dice coefficient proposed in Milletari et al. (2016) in this study. The loss ***L*** is defined as follows:

(3) $$\begin{array}{l} \text{L}=1-\frac{2\sum_{i=1}^{N}{\text{s}}_{i}{\text{g}}_{i}}{\sum_{i=1}^{N}{\text{s}}_{i}^{2}+\sum_{i=1}^{N}{\text{g}}_{i}^{2}} \end{array}$$

where *N* is the number of voxels, ***s***_*i*_ and ***g***_*i*_ belong to the binary segmentation and binary ground truth voxel sets, respectively. The loss function measures the similarity of two samples directly.

### 3.5. Liver Localization Using RA-UNet-I

The first stage aimed to locate the 3D liver boundary box. A 2D version RA-UNet-I was introduced here to segment a coarse liver region, which can reduce the computational cost of the subsequent RA-UNet-II, remove the redundant information, and provide more effective information. It worked as a “baseline” to limit the scope of the liver.

We down sampled the slices to 256×256 and fed the preprocessed slices into the trained RA-UNet-I model. Next, we stacked all the slices in their original sequence. Afterwards, a 3D connected-component labeling (Hossam et al., 2010) was employed. The connected component labeling, which is used for determining specific regions and measure the size of regions, is a procedure for assigning a unique label to each connected component in an image. Then the largest component was chosen as the coarse liver region. Finally, we interpolated the liver region to its original volume size with a 512 × 512 in-plane resolution.

Connected component labeling is a procedure for assigning a unique label to each connected component in an image.

### 3.6. Liver Segmentation Using RA-UNet-II

The RA-UNet-II was a 3D model which fully utilized the volume information and captured the spatial information. The 3D U-Net type architecture (Çiçek et al., 2016) merges the low resolution and high resolution features to generate an accurate segmentation. Meanwhile, using large image patches (224 × 224 × 32) for training provides much richer contextual information than using small image patches, which usually leads to more global segmentation results.

As shown in Table 2, the network went down from the top to the bottom in the encoder, and reversed in the decoder. During the encoding phase, the RA-UNet-II received liver patches and passed them down to the bottom. During the decoding phase, lower features were passed from the bottom to the top with resolution doubled through the up-sampling operation. Note that the long-range connection between the encoder and the decoder was realized by the attention block. We then combined the features from the attention blocks with those from the corresponding up-sampling level in the decoder via concatenation. Then the concatenated features were passed on to the decoder. Finally, an activation layer (i.e., Sigmoid) was used to generate the final probability map of liver segmentation.

**Table 2:** Architecture of the proposed RA-UNET-II in liver localization stage.

**Encoder**	**Output size**	**Decoder**	**Pre-operation**	**Output size**
Input	224 × 224 × 32 × 1	Att1	[Res4], depth=0	14 × 14 × 2 × 256
Conv1	224 × 224 × 32 × 32	Res7	[Up1, Att1]	14 × 14 × 2 × 256
Pooling	112 × 112 × 16 × 32	Up2		28 × 28 × 4 × 256
Res1	112 × 112 × 16 × 32	Att2	[Res3], depth=1	28 × 28 × 4 × 128
Pooling	56 × 56 × 8 × 32	Res8	[Up2, Att2]	28 × 28 × 4 × 128
Res2	56 × 56 × 8 × 64	Up3		56 × 56 × 8 × 128
Pooling	56 × 56 × 4 × 64	Att3	[Res2], depth=2	56 × 56 × 8 × 64
Res3	28 × 28 × 4 × 128	Res9	[Up3, Att3]	56 × 56 × 8 × 64
Pooling	14 × 14 × 2 × 128	Up4		112 × 112 × 16 × 64
Res4	14 × 14 × 2 × 256	Att4	[Res1], depth=3	112 × 112 × 16 × 32
Pooling	7 × 7 × 1 × 256	Res10	[Up4, Att4]	112 × 112 × 16 × 32
Res5	7 × 7 × 1 × 512	Up5		224 × 224 × 32 × 32
Res6	7 × 7 × 1 × 512	Conv2	[Up5, Conv1]	224 × 224 × 32 × 32
Up1	14 × 14 × 2 × 512	Conv3		224 × 224 × 32 × 1

*Here [ ], long range connection; [,], concatenate operation; Conv, convolution; Up, up-sampling; Res, residual block; Att, attention block*.

The RA-UNet-II has fewer parameters than the traditional U-Net (Ronneberger et al., 2015). With this architecture, the number of parameters has been largely decreased to only 4M training parameters. During the training phase, we interpolated the liver boundary box in the *x*−*y* plane to a fixed size (i.e., 224×224) and randomly picked 32 slices successively in the *z* direction to form the training patches. The RA-UNet-II was employed on each CT patch to generate 3D liver probability patches in sequence. Then, we interpolated and stacked those probability patches to be restored to the original size of the boundary box. A voting strategy was used to generate the final liver probability of the VOI from overlapped sub-patches. A 3D connected-component labeling was used and the largest component was chosen on the merged VOI to yield the final liver region.

### 3.7. Extraction of Tumors Based on RA-UNet-II

**Figure 6:**
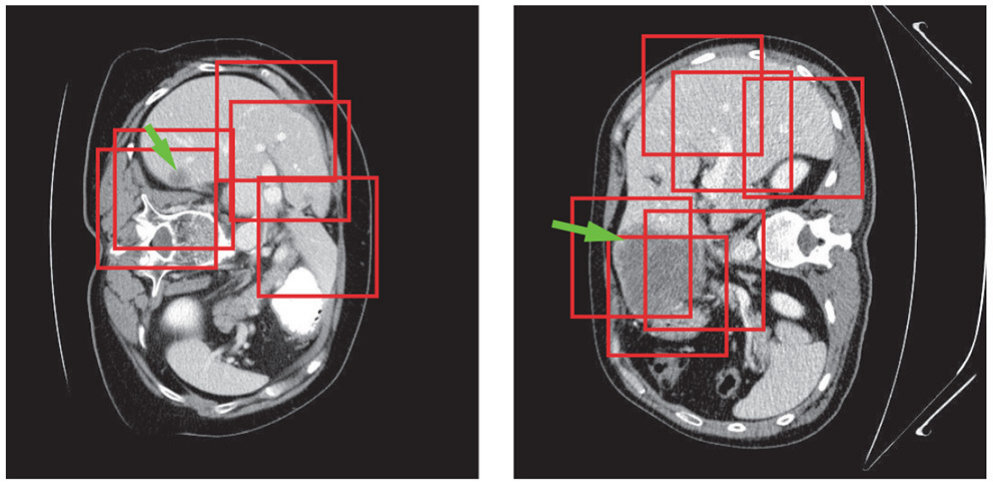
Tumor patch extraction results. The green arrows point to the tumor regions and the red boxes show the patches used for training.

Tumor region extraction was similar to liver segmentation but no interpolation and resizing were performed. Because the size of the tumor is much smaller than that of the liver, the original tumor resolution was used to avoid losing small lesions. Furthermore, in order to solve the data imbalance issue and learn more effective tumor features, we picked patches on both tumor and its surroundings non-tumor regions for training as shown in Figure 6. Note that only those in the liver VOIs would be the candidate patches for training. We extracted the tumors following a similar routine as for the liver segmentation step except the use of interpolation. Subsequently, a voting strategy is used again on the merged VOI to yield the final tumor segmentation. At last, we filtered out those voxels which were not in the liver region.

### 3.8. Evaluation Metrics

We evaluated the performance of the proposed approach using the metrics introduced in Heimann et al. (2009). The evaluation metrics include the Dice score (DS) (Wu et al., 2016) consist of Dice global (Dice score computed on all combined volumes denoted with DG) and Dice per case (mean Dice score per volume denoted with DC), Jaccard similarity coefficient (Jaccard), volumetric overlap error (VOE), relative volume difference (RVD), average symmetric surface distance (ASSD), and maximum surface distance (MSD).

### 3.9. Implementation Details

The RA-UNet architecture was constructed using the Keras (Chollet, 2015) and the TensorFlow (Abadi et al., 2015) libraries. All the models were trained from scratch. The parameters of the network were initialized with random values and then they were trained with back-propagation based on Adam (Kingma and Ba, 2014) with an initial learning rate (LR) of 0.001, β_1_=0.9, and β_2_=0.999. The learning rate would be reduced to LR×0.1 if the network went to plateau after 20 epoches. We used 5-fold cross-training on the LiTS training dataset, and evaluated the performance on the LiTS test dataset. To demonstrate the generalization of our RA-UNet, we also evaluated the performance on the 3DIRCADb dataset using the well-trained weights from the LiTS training dataset. For the liver and tumor training, the total numbers of epoches were set at 50 and 50 for each fold, respectively. An integration operation by a voting strategy is implemented to ensemble all the prediction results of 5 models. The training of all the models was performed with an NVIDIA 1080Ti GPU. In our experiments, it took about 100/40 min to train an epoch of our 3D RAUNet for liver/tumor segmentation, respectively.

## 4. Experiments and Results

### 4.1. Liver Volume of Interest Localization

In order to reduce the computational cost, we first down-sampled the input slices to a 256 × 256 pixel in-plane resolution. Secondly, we used all the slices which have liver in the images together with 1/3 of those randomly picked slices without liver as the training data. There are a total of 32,746 slices with liver which were used, including 23,283 slices for training and 9,463 slices for validation. Note that 5-fold training was not employed at this stage, because our goal at this stage was to obtain a coarse liver boundary box and reduce the computational time.

**Figure 7:**
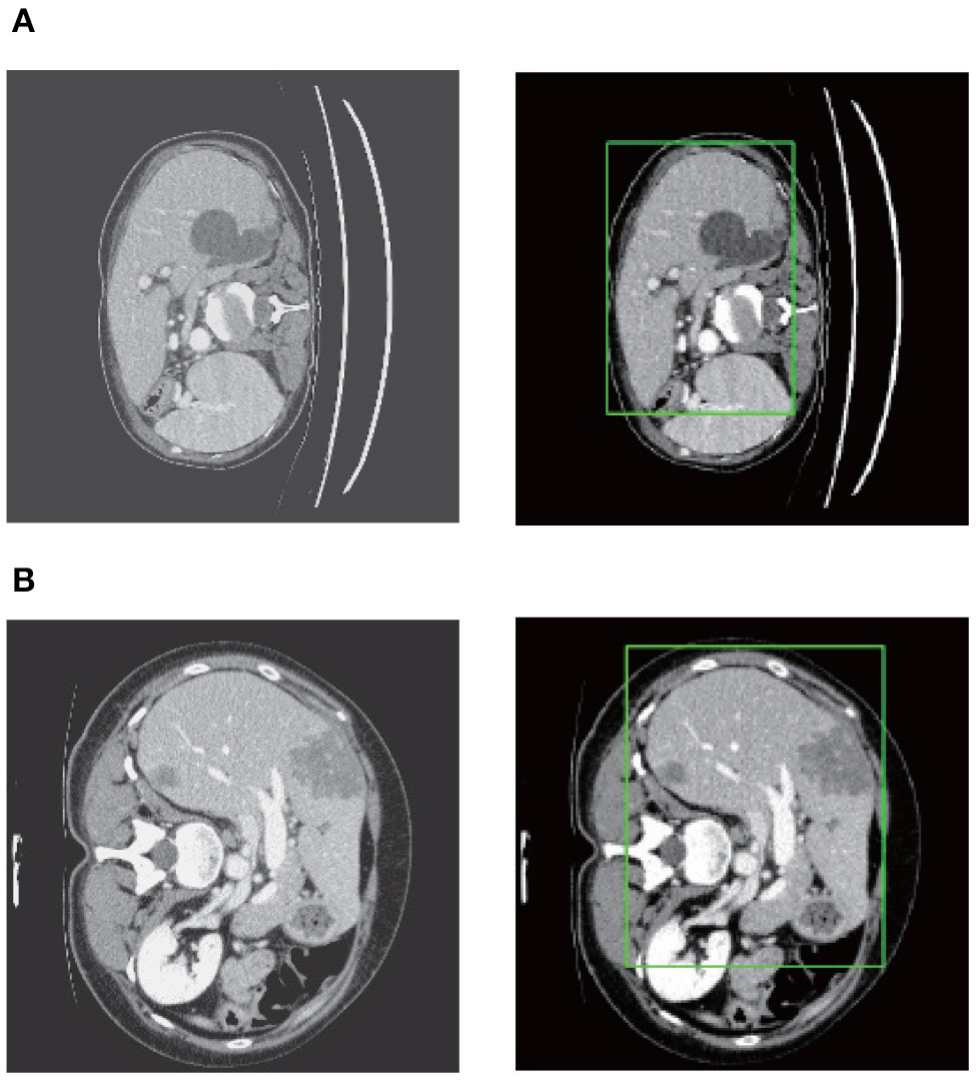
Liver localization using RA-UNet-I. From left to right the figure shows the preprocessed slice, and the final boundary box which restricts the liver region. **(A)** A typical slice from the LiTS validation dataset. **(B)** A typical slice from the 3DIRCADb dataset. The RA-UNet-I enables the coarse localization of liver regions.

After stacking all the slices and employing the 3D connected-component labeling, we calculated the 3D boundary box of the slices with liver, and extended 10 pixels in coronal, sagittal, and axial directions to ensure that the entire liver region was included. Figure 7 shows the liver localization results from RA-UNet-I. It demonstrates that the attention mechanism has successfully constrained the liver region. Note that this stage aims to reduce the computational cost for precisely segmenting liver and tumor by RA-UNet-II.

### 4.2. Liver Segmentation Using RA-UNet-II

RA-UNet-II allowed the network to go “deeper.” However, the implementation of a 3D convolution is still limited by the hardware and memory requirements (Prasoon et al., 2013). In order to balance the computational cost and efficiency, we first carried out interpolation in the region inside the liver boundary box to the size of 224 × 224 × *M*, where *M* was the axial length of the liver boundary box. Then we cropped the volumetric patches (224 × 224 × 32) randomly from each boundary box, which was constrained by the liver boundary box. Totally, 4,077/1,019 patches were selected for training/validation.

**Figure 8:**
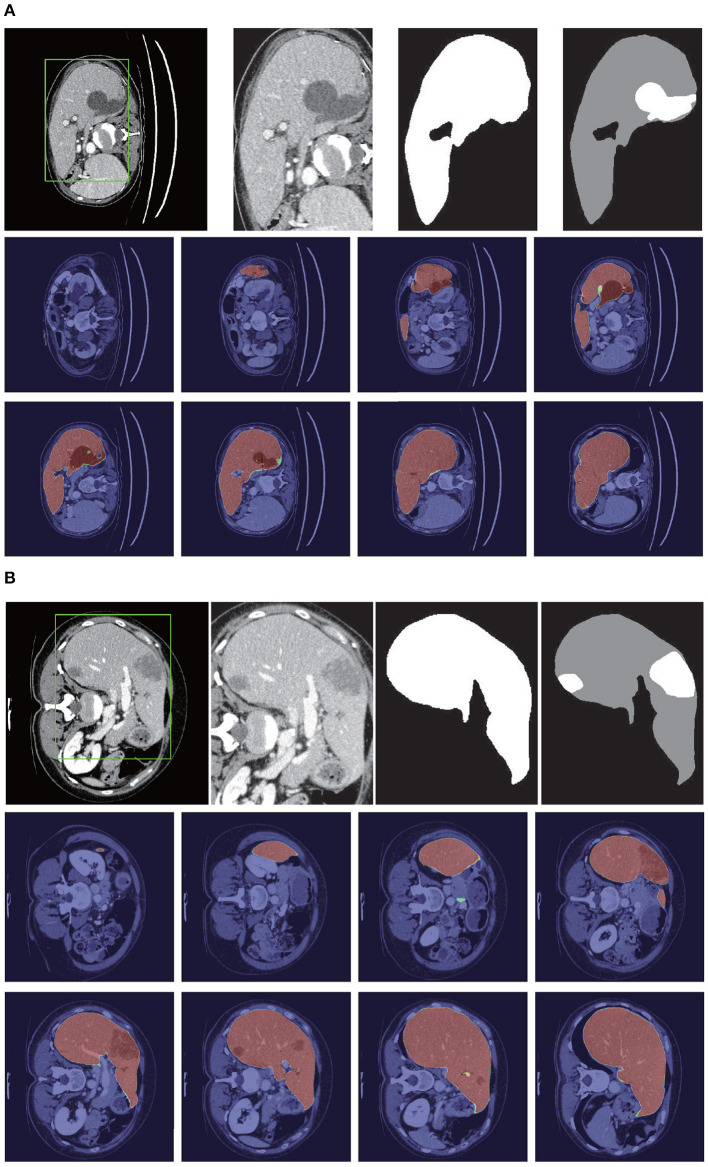
Liver segmentation results based on RA-UNet-II. **(A)** From the LiTS validation dataset and **(B)** is from the 3DIRCADb dataset. From left to right, the first row of each subplot shows the liver in the green boundary box, magnified liver region, the liver segmentation results, and the corresponding ground truth. The second and the third rows show the probability heat map of liver segmentation results. The darker the color, the higher the probability of the liver region. Note that the ground truth contains liver in gray and tumor in white.

Figure 8 shows the liver segmentation based on RA-UNet-II, which indicates that our proposed network has the ability to learn 3D contextual information and could successfully extract the liver from adjacent slices in an image volume. After the 3D connected-component labeling was carried out, the liver region was precisely extracted by selecting the largest region.

As shown in Table 3, our method reached up to 0.961 and 0.977 Dice scores on the LiTS test dataset and the 3DIRCADb dataset, respectively. It reveals that RA-UNet yields remarkable liver segmentation results. Then we can extract tumors from the segmented liver regions.

**Table 3:** Evaluation results of the liver segmentation on the LiTS test dataset and the 3DIRCADb dataset.

	**LiTS**	**3DIRCADb**
DC	0.961	0.977
Jaccard	0.926	0.977
VOE	0.074	0.045
RVD	0.002	−0.001
ASSD	1.214	0.587
MSD	26.948	18.617

### 4.3. Extraction of Tumors Based on RA-UNet-II

**Figure 9:**
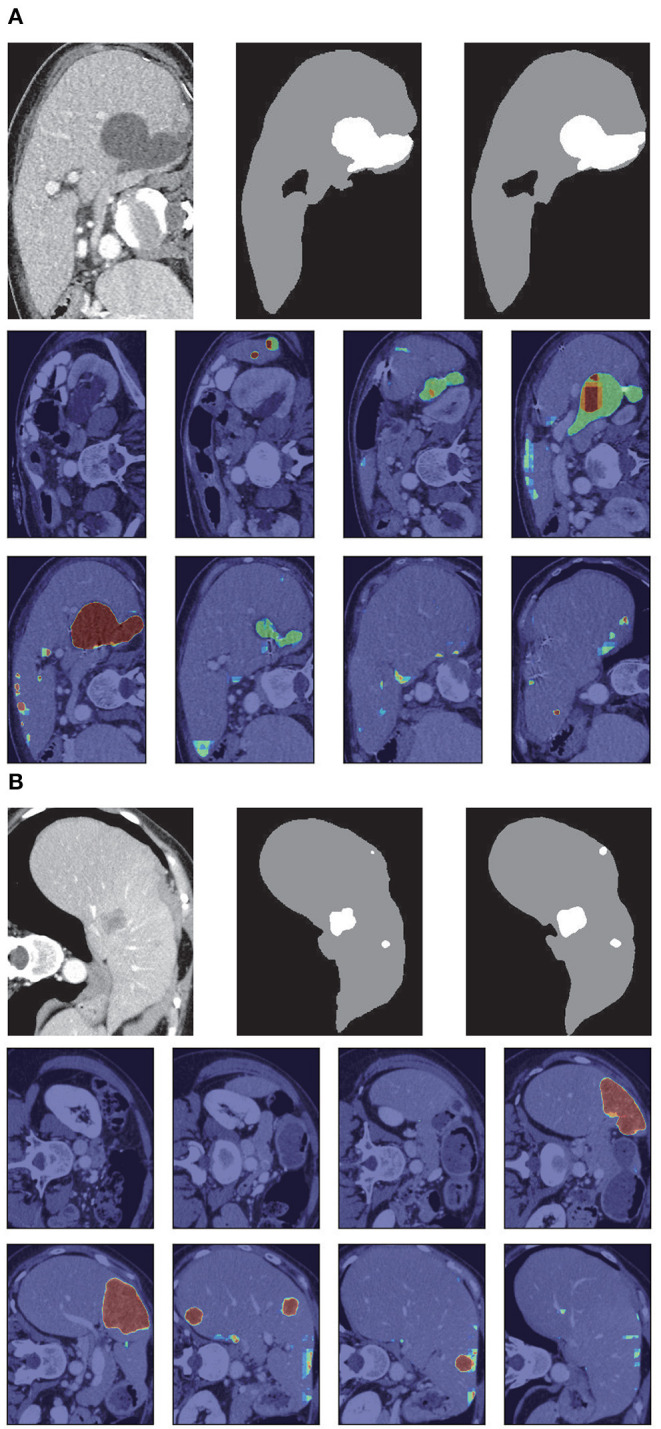
Tumor segmentation results based on RA-UNet-II. **(A)** From the LiTS validation dataset, and **(B)** is from the 3DIRCADb dataset. From left to right, the first row of each subplots indicates the raw images, segmentation results of liver tumor, and the corresponding ground truth. The second and the third rows show the probability heat map of tumor segmentation results.

Tumors were tiny structures compared to livers. Therefore, no interpolation or resizing was applied to tumor patch sampling to avoid information loss from image scaling. It was difficult to decide what size of patch for training could reach a desirable performance. In order to determine the patch size, we set the patch size of 32×32×32, 64×64×32, and 128×128×32, respectively to test the performance of tumor segmentation. Results showed that 128×128×32 patch-sized data achieved the best tumor segmentation performance. The larger the patch size was, the richer context in formation the patches could provide. Due to the limitation of computational resources, 128×128×32 was chosen empirically for tumor patches. We randomly picked 150 patches from each liver volume in the boundary box. Totally, 14,160/3,540 patches were chosen from LiTS as training/validation datasets. As shown in Table 4, our method reached 0.595 and 0.830 Dice scores on the LiTS test dataset and the 3DIRCADb dataset, respectively. Figure 9 shows the tumor segmentation results in detail.

**Table 4:** Scores of the tumor segmentation on the LiTS test dataset and the 3DIRCADb dataset.

	**LiTS**	**3DIRCADb**
DC	0.595	0.830
Jaccard	0.611	0.744
VOE	0.389	0.255
RVD	−0.152	0.740
ASSD	1.289	2.230
MSD	6.775	53.324

**Figure 10:**
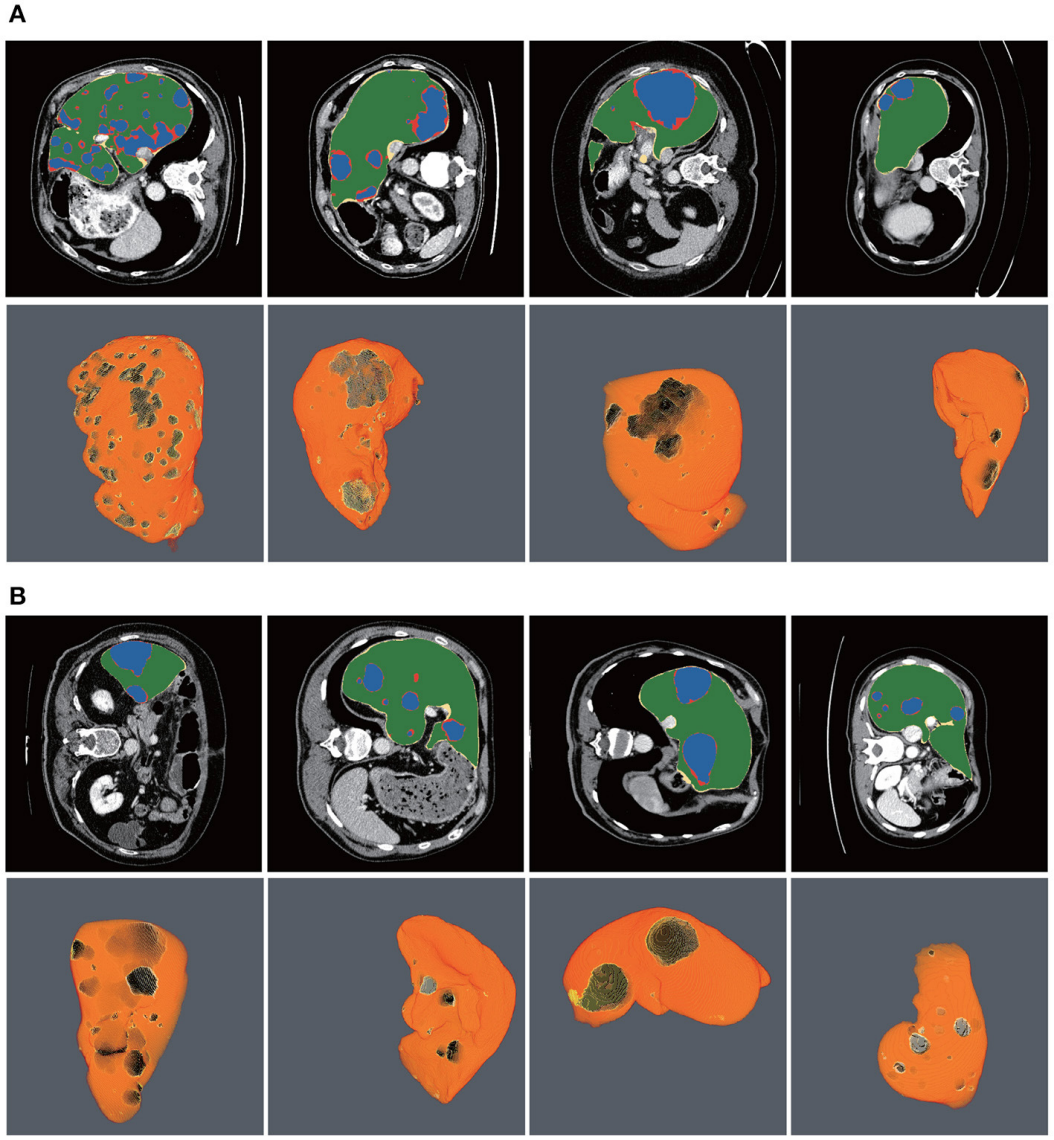
Automatic liver and tumor segmentation with RA-UNet. The green regions indicate the correctly extracted liver, the yellow regions are the wrongly extracted liver, the blue color depicts the correctly extracted tumor regions, and the red color means wrongly extracted tumor. The first row of each subplot shows four slices from different volumes in the axial view and the second row of each subplot shows the corresponding 3D view of the entire liver/tumor segmentation results. **(A)** From the LiTS dataset. **(B)** From the 3DIRCADb dataset.

Figure 10 shows the liver/tumor segmentation results. It shows that liver regions which are large in size are successfully segmented and tumors that are tiny and hard to detect can be identified by the proposed method as well. Due to the low contrast with the surrounding livers and the extremely small size of some tumors, the proposed method still has some false positives and false negatives for tumor extraction.

### 4.4. Comparison With Other Methods

There were several submissions about liver and tumor segmentations to the 2017 ISBI and MICCAI LiTS challenges. We reached a Dice per case of 0.961, Dice global of 0.963, Jaccard of 0.926, VOE of 0.074, RVD of 0.002, ASSD of 1.214, and MSD of 26.948, which is a desirable performance on the LiTS challenge for liver segmentation. For tumor segmentation evaluation, our method reached a Dice per case of 0.595, Dice global of 0.795, Jaccard of 0.611, VOE of 0.389, RVD of −0.152, ASSD of 1.289, and MSD of 6.775. Compared with other methods, Bellver et al. (2017) and Pandey et al. (2018) methods reached tumor Dice per case at 0.587 and 0.59, respectively, which were 2D segmentation methods. Our approach outperformed these two methods. The detailed results and all the performances are listed in Table 5. It is worth mentioning that our method for precise segmentation of liver and tumor was a full 3D technique with a much deeper network.

**Table 5:** Segmentation results compared with other methods on the LiTS test dataset.

		**LiTS liver**							**LiTS tumor**						
	**Dimension**	**DC**	**DG**	**Jaccard**	**VOE**	**RVD**	**ASSD**	**MSD**	**DC**	**DG**	**Jaccard**	**VOE**	**RVD**	**ASSD**	**MSD**
Kaluva et al. (2018)	2D	0.912	0.923	0.850	0.150	−0.008	6.465	45.928	0.492	0.625	0.589	0.411	19.705	1.441	7.515
Bi et al. (2017)	2D	0.959	–	0.922	–	–	–	–	0.500	–	0.388	–	–	–	–
Li et al. (2018)	2.5D	0.961	0.965	–	0.074	−0.018	1.450	27.118	0.722	0.824	–	0.366	4.272	1.102	6.228
MEDDIIR	Unknown	0.950	0.955	–	0.094	0.047	1.597	28.911	0.658	0.819	–	0.380	−0.129	1.113	6.323
Yuan (2017)	2D	0.963	0.967	–	0.071	−0.010	1.104	23.847	0.657	0.820	–	0.378	0.288	1.151	6.269
Summer	Unknown	0.941	0.945	–	0.108	−0.066	6.552	152.350	0.631	0.786	–	0.400	−0.181	1.184	6.367
Proposed method	3D	0.961	0.963	0.926	0.074	0.002	1.214	26.948	0.595	0.795	0.611	0.389	−0.152	1.289	6.775

### 4.5. Generalization of the Proposed RA-UNet

To show the generalization of the proposed method, we used the weights well-trained on LiTS and tested on the 3DIRCADb dataset. Some works concentrated on liver segmentation, and there were a few about tumor segmentation. Hence, we listed the results of some approaches in Table 6. Our methods reached a Dice per case of 0.977, Jaccard of 0.977, VOE of 0.045, RVD of −0.001, ASSD of 0.587, and MSD of 18.617, which quantitatively show that our method performed significantly better than all the other methods on liver segmentation. Since most of the works aimed at liver segmentation, few of them displayed tumor segmentation results, we only compared with Christ et al. (2017a) on the 3DIRCADb dataset. It was worth mentioning that our method reached a mean Dice score of 0.830 on livers with tumors compared to a mean Dice score of 0.56 for the method by Christ et al. (2017a). The visualization of typical performance was illustrated in Figures 8B, 9B, 10B, which qualitatively indicated that our method produced precise segmentation performance.

**Table 6:** Segmentation results compared with other methods on the 3DIRCADb dataset.

		**3DIRCADb liver**						**3DIRCADb tumor**
	**Dimension**	**DC**	**Jaccard**	**VOE**	**RVD**	**ASSD**	**MSD**	**DC**
Christ et al. (2017a)	2D	0.943	–	0.107	−0.014	1.6	24	0.56
Ronneberger et al. (2015)	2D	0.729	–	0.39	0.87	19.4	119	–
Li et al. (2013)	2D	0.945	–	0.068	−0.112	1.6	28.2	–
Eapen et al. (2015)	3D	–	–	0.0554	0.0093	0.78	15.6	–
Lu et al. (2017)	3D	–	–	0.0936	0.0097	1.89	33.14	–
Proposed method	3D	0.977	0.977	0.045	−0.001	0.587	18.617	0.83

## 5. Conclusion

To summarize our work, we have proposed an effective and efficient hybrid architecture for automatic extraction of liver and tumor from CT volumes. We introduce a new 3D residual attention-aware liver and tumor segmentation neural network named RA-UNet, which allows the extraction of 3D structures in a pixel-to-pixel fashion. The proposed network takes advantage of the strengths from the U-Net, the residual learning, and the attention residual mechanism. Firstly, attention-aware features change adaptively with the use of attention modules. Secondly, the residual blocks are stacked into our architecture which allows the architecture to go deeply and solve the gradient vanishing problem. Finally, the U-Net is used to capture multi-scale attention information and integrate low-level features with high-level features. To the best of our knowledge, this is the full 3D model and the first time that the attention residual mechanism is implemented in the medical imaging tasks. Fewer parameters are trained by the attention residual mechanism. The proposed method enlarges the U-Net family for 3D liver and tumor segmentation tasks, which is crucial for real-world applications. The effective system includes three stages: liver localization by the RA-UNet-I, precise segmentation of liver, and tumor lesion by the RA-UNet-II. More importantly, the trained network is a general segmentation model working on both the LiTS and the 3DIRCADb datasets.

Overall, our method achieved competitive performances in liver tumor challenge, and exhibits high extension and generalization ability in another tumor segmentation dataset. The proposed model has great potential to be applied to other modalities of medical images. It may also assist surgeons to find treatment for novel tumors. The limitation of the proposed method is the training time because the 3D convolutions require larger parameters than the 2D convolutions. In future work, we aim to further improve the architecture, making the architecture much more general to other tumor segmentation datasets and more flexible to common medical imaging tasks. What's more, reducing computational cost and developing a lightweight architecture for speeding training time are also under consideration.

## Data Availability Statement

Publicly available datasets were analyzed in this study. This data can be found at: https://competitions.codalab.org/competitions/17094.

## Author Contributions

QJ conducted the experiments. ZM, CS, and HC participated in manuscript writing. RS designed the experiments and edited the manuscript. All authors contributed to the article and approved the submitted version.

## Conflict of Interest

The authors declare that the research was conducted in the absence of any commercial or financial relationships that could be construed as a potential conflict of interest.

## Footnotes

^1^https://github.com/RanSuLab/RAUNet-tumor-segmentation.git
